# Supplementary material for: Spontaneous slow cortical potentials and brain oscillations independently influence conscious visual perception
Source: PLoS Biol. 2025 Jan 16;23(1):e3002964. doi: 10.1371/journal.pbio.3002964 (PMC11737857; doi:10.1371/journal.pbio.3002964)
Supplement: S1 Text — (DOCX) [file pbio.3002964.s001.docx]

Fig A:

**SUPPLEMENTARY FIGURE: Simulation to control for time-domain smearing.**

**
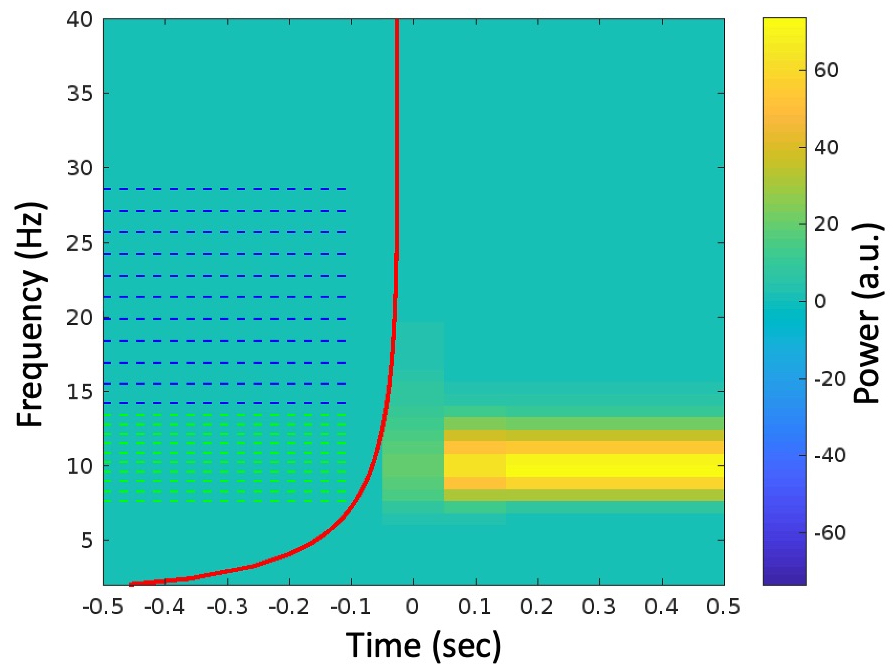
**

Time-frequency plot of a simulated 10 Hz sinusoidal wave using wavelet power-estimation parameters identical to those used in all analyses (i.e., frequency resolution of 0.8 Hz, temporal resolution of 0.1 s, wavelet width linearly increasing with frequency from 3 to 9 cycles). The red curved line indicates the time points before which oscillatory activity is not influenced by the post-stimulus 10 Hz signal. The hatched green area indicates the alpha band on the y-axis, and the hatched blue area indicates the beta band on the y-axis, and both are bounded at -0.1 s, such that they are not contaminated by any post-stimulus signal.

Note A

**SUPPLEMENTARY NOTE: Details of the time-frequency analysis pipeline**

In this Supplementary Note, we describe the detailed steps of our analysis to extract the oscillation power on each trial while separating the oscillations from the 1/f power spectrum.

1) We first pre-process our MEG data, and from the continuous time course, we obtain 4-second-long epochs that range -2 seconds to +2 seconds around stimulus onset. We only investigated the pre-stimulus range from -1.7 sec onward to avoid any edge effects in the time-frequency estimation. Plotted below if an example epoch from one subject.


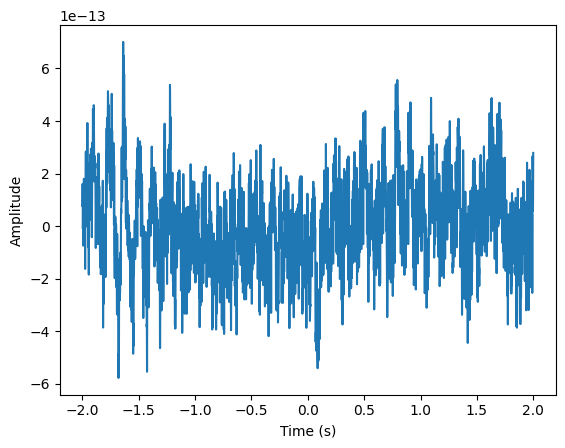


2) To obtain power changes in the alpha and beta frequency bands related to the detection (low-level dataset) or recognition (high-level dataset) of stimuli, we take the continuous data from -1.7 s to 0 s, and then apply a wavelet transform (Morlet wavelets, 47 frequencies, frequency range: 0.8 ­– 40 Hz, number of cycles increasing linearly from 3 to 9, time window: -1.7 to -0.1 s relative to stimulus onset) to the MEG time courses. We obtain power spectra with 0.8 Hz frequency resolution, for every 100 ms interval of the data. We therefore obtain 17 power spectra in the pre-stimulus interval (across 1.7 s of data, one estimate every 100 ms). Below, we plot the power spectrum for one subject, for four 100 ms intervals in the pre-stimulus interval of a single trial. Here, the power spectra are plotted for the -1.7 s, the -1.6 s, the -1.5 s and the -1.4 s intervals, in blue, red, orange, and green respectively.

3) Given that we are estimating power spectra on single-trial data, we want our spectral parametrization, estimated by fooof, to be as good as possible, and based on our tests, these estimates are best if the input signal has the lowest signal-to-noise ratio. By averaging four 100 ms power spectra estimates together, we obtain higher signal-to-noise ratio. In the plot below, the thick black line shows the averaged power spectrum across the four wavelet-estimated power spectra for the first four 100 ms time intervals, which was input into fooof.


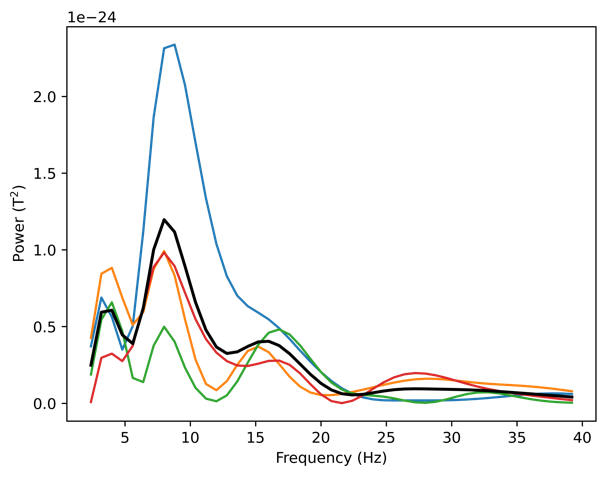


4) Then, we use these 400-ms averaged power spectra as input for the fooof algorithm, which performs the spectral parametrization. For the trial and temporal interval shown above, the fooof algorithm performs the following fit:


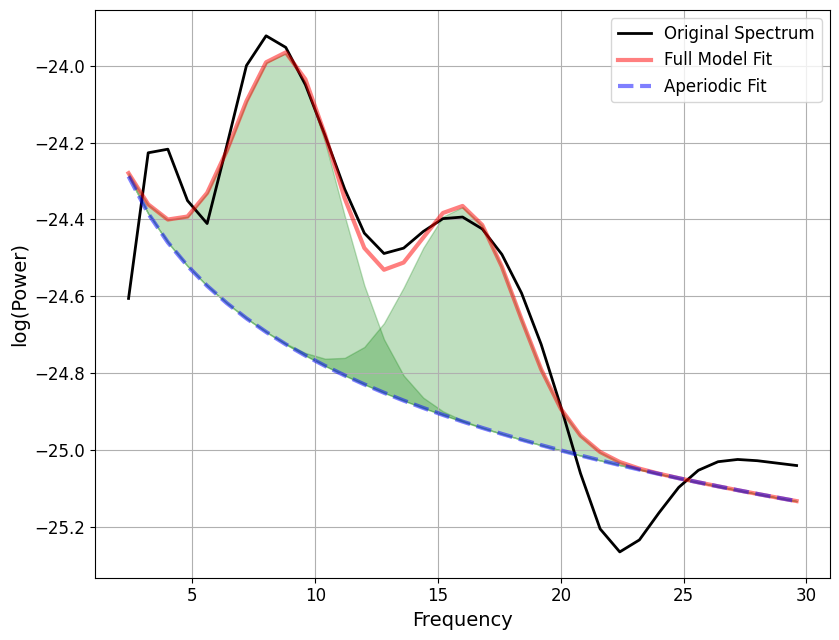


In this fit, the green shaded areas correspond to different oscillatory peaks identified by the algorithm (one alpha peak, and one beta peak).

5) Within the alpha and the beta band, if fooof has identified multiple peaks, we select a single peak that has the highest power.

6) To obtain the power of alpha and beta oscillations, we first convert the aperiodic fit and the full model fit into linear space. We then compute the area under the curve (AUC) from one bandwidth (${BW}_{\alpha} or {BW}_{\beta}$) below the center frequency of the detected oscillation (${CF}_{\alpha} or {CF}_{\beta})$ to one bandwidth above it, as follows:

$${AUC}_{\alpha}= \sum_{{CF}_{\alpha}- {BW}_{\alpha}}^{{CF}_{\alpha}+ {BW}_{\alpha}} total- \sum_{{CF}_{\alpha}- {BW}_{\alpha}}^{{CF}_{\alpha}+ {BW}_{\alpha}} aperiodic$$

$${AUC}_{\beta}= \sum_{{CF}_{\beta}- {BW}_{\beta}}^{{CF}_{\beta}+ {BW}_{\beta}} total- \sum_{{CF}_{\beta}- {BW}_{\beta}}^{{CF}_{\beta}+ {BW}_{\beta}} aperiodic$$

For both equations, *total* is the total linear spectrum (or full model fit), including both aperiodic fit and oscillations, and *aperiodic* is the aperiodic spectrum. See **Fig. 1F** (left panel) for an illustration of the full decomposition. Below we plot the linear aperiodic fit in dashed blue line and the linear full model fit in red. The dotted red line indicates the center frequency (CF) of the oscillation of interest (in this case, an alpha oscillation with CF = 8.8 Hz), and the dashed black arrow indicates its bandwidth (BW), which corresponds to full-width-at-half-maximum (FWHM). The green dotted lines indicate the range across which we sum the linear fits, corresponding to the frequency range from CF–BW to CF+BW. We chose this range because *fooof* outputs the bandwidth as FWHM, which corresponds to only the upper half of the oscillatory peak. The orange shaded area corresponds to the AUC for the aperiodic fit and is obtained by summing the linear *aperiodic* fit between the two dotted green lines. The yellow shaded area and orange shaded area together corresponds to the AUC for the full model fit and is obtained by summing the linear *full model* fit between the two dashed lines. The power (AUC) for the oscillatory peak is thus obtained by subtracting the AUC for the aperiodic fit from the AUC for the full model fit and corresponds to the yellow shaded area above the aperiodic fit. Thus, the subtraction between full power and aperiodic power was performed in the linear space, consistent with the assumption of an additive relationship between the aperiodic activity and oscillatory activity, and correspondingly, an additive relationship between their power (*1, 2*).


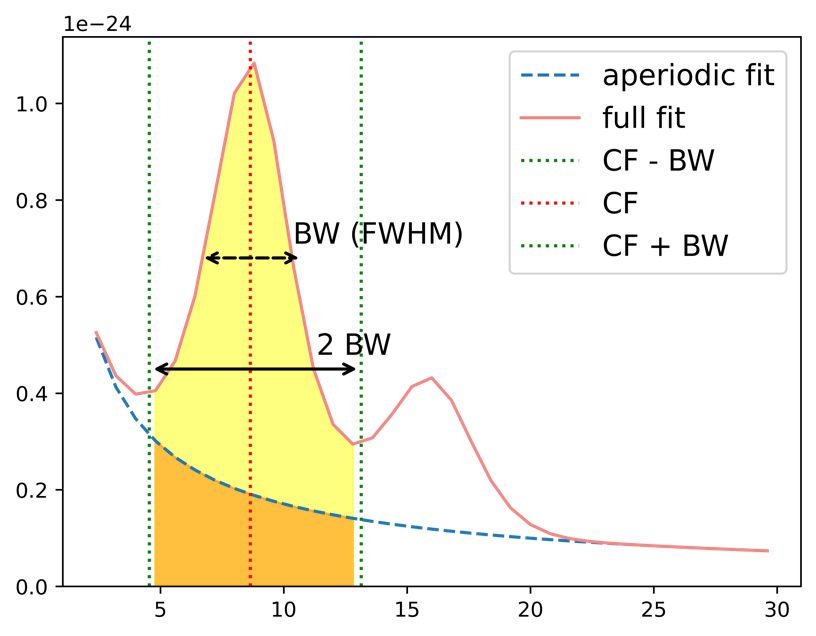


References:

1. M. Gyurkovics, G. M. Clements, K. A. Low, M. Fabiani, G. Gratton, The impact of 1/f activity and baseline correction on the results and interpretation of time-frequency analyses of EEG/MEG data: A cautionary tale. Neuroimage 237, 118192 (2021).

2. B. J. He, J. M. Zempel, A. Z. Snyder, M. E. Raichle, The temporal structures and functional significance of scale-free brain activity. Neuron 66, 353-369 (2010).
