# Supplementary material for: Spontaneous slow cortical potentials and brain oscillations independently influence conscious visual perception
Source: PLoS Biol. 2025 Jan 16;23(1):e3002964. doi: 10.1371/journal.pbio.3002964 (PMC11737857; doi:10.1371/journal.pbio.3002964)
Supplement: Attachment — Submitted filename: Koenig rebuttal letter 20241002.pdf [file pbio.3002964.s002.pdf]

We would like to thank all three reviewers for the careful review and constructive feedback. In what follows, we provide point-by-point reply to the reviewers' comments in black, with the original text from the reviewers in blue.

Reviewer #1 (Afra Wohlschläger):

The manuscript presents an interesting reanalysis of two MEG-datasets on upcoming visual perception on lower-level and higher-level visual stimuli, respectively. The influence of SCPs on perception is directly compared to the influence exerted by the oscillatory activities within the alpha- and beta-bands. The study finds significant but independent contributions to confidence in visual perception. For one study the relationship towards the noradrenergic system is additionally studied via the pupil diameter. This adds important insights on the mechanistic level. The manuscript is well written and describing interesting and well-motivated analyses.

I have few concerns:

1) Why are data on all stimuli used, i.e. why weren't catch trials omitted? Shouldn't this alter the interpretation in that prediction of confidence is assessed rather than perceptual success? The title should be: 'Spontaneous slow cortical potentials and brain oscillations independently influence confidence in visual perception' Or alternatively a discussion of this aspect should be extended.

We thank the reviewer for raising this relevant point. We agree that the text should clarify our motivation for using all trial types, including catch trials.

Our initial rationale for including catch trials was that recent research indicates that fluctuations in pre-stimulus alpha power (*1*) and slow cortical potentials (SCPs) (*2, 3*) are associated with changes in the perceptual criterion. In fact, the observation that both alpha power and SCPs predict trial-to-trial criterion shift in threshold-level perception tasks was an important motivation for the present study probing whether they have shared mechanisms or act independently. The criterion is computed by including perceptual reports from both 'real' and catch trials. We aimed to build on these findings and examine whether pre-stimulus neural signals related to changes in the criterion were independent or interdependent. Additionally, given that we chose to focus on single-trial estimates of these neural signals, we analyzed both 'real' and catch trials (rather than probing criterion effects directly, which would require pooling these trials together and hence losing trial-wise measures of neural activity).

Whether catch trials are included or not, we believe that our analyses allow us to draw conclusions about perceptual awareness rather than confidence, in line with research showing that perceptual awareness and confidence can be dissociated (*4, 5*). When a participant reported having perceived a stimulus, whether about the presence of a stimulus (low-level dataset) or about the presence of a meaningful content (high-level dataset), their report related to their conscious experience of the stimulus and could be associated with both low or high confidence. In other words, these reports relate to 1st order subjective perceptions, while confidence reports are higher-order metacognitive judgments. In fact, using the low-level data set, we have previously shown that neural activity related to perceptual awareness, as assessed by the "seen" vs. "unseen" response, can be strongly dissociated from neural activity related to metacognitive confidence judgments (*6*).

Finally, on a more technical point, we show below that the decoder for classifying perceived vs. unperceived trials in all trials is very similar to the decoder for classifying perceived vs. unperceived in only real trials (i.e., excluding catch trials). Using the high-level dataset, we extracted the decision axis of each

decoder for each subject and pre-stimulus timepoint. We then computed the angle between the two decision axes and plot the mean ± sem across subjects for each timepoint. If the decoders are similar, the angle between their decision axes will be slight. We further plot two control time courses: during the decoding cross-validation, given that we run ten repetitions (see *Methods* for details), we computed the angle between the mean decision axis across the first five repeats and the mean decision axis across the second five repeats. This gives us an estimate of the noise level for the decoder. We do this for both the decoder using all trials and for the decoder using only real trials. By comparing the time courses, we see that the decision axes for decoding recognition in 'all trials' versus in only 'real trials' are very similar because the angle between them is relatively small (~30°) and not much larger than for the control time courses (~20°). We ran this control analysis in the high-level data set, which has a higher percentage (16.7%) of catch trials than the low-level dataset (~5%), so the difference between decoders trained on all trials vs. all real trials is expected to be even smaller in the low-level data set.

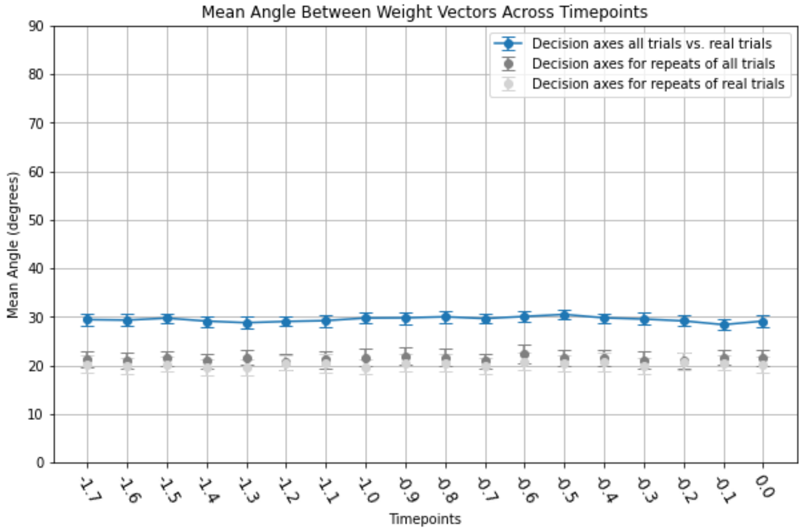

Consistent with these observations, our previous study using the high-level data set (*3*) showed that a decoder trained on catch trials to predict "recognized" vs. "unrecognized" responses had significant above-chance decoding accuracy when tested on real trials, and vice versa (see Fig. 2A of that paper).

We have now added a sentence to our Results section on p.4 explaining the rationale for including all trials.

2) The analysis strategies differ between the two datasets with regards to the SCPs. I have related questions and remarks.

Methods section: Decoding analysis and Fig 2: How does the time axis in Fig. 2 A and D come about? The methods for deriving the d.v. are considerably different between the two data sets. From how I understand the analyses, for the low-level dataset there should be 1 d.v. value prior to the stimuli and none after, for the high-level dataset there should be four values prior to the stimuli and none after. It should be made clear that Fig 1E only depicts the strategy for the lower-level dataset. Otherwise, the figures overall mimic identical strategies which is not the case.

We thank the reviewer for this question and apologize for the confusion. In fact, the methods for deriving the SCP d.v. are almost identical between the two datasets; the biggest difference is the type of decoder.

That is, while the low-level dataset decoding is conducted using a support vector machine (SVM), the high-level dataset decoding is done with logistic regression. We don't believe that this choice of the decoder would make a tangible difference in the results, because in those prior studies we had found that the results were robust to the specific choice of the decoder in preliminary analysis. In ref (*3*), we chose to use logistic regression classifier because it allowed an intuitive interpretation based on probability that is better connected with our hypotheses (see Methods section therein). Here, to facilitate continuity and easier comparison with prior papers, we chose to use the same decoders as used in the prior publications.

For each of these datasets, data were first down sampled to 10 Hz, and we extracted a SCP d.v. value for each trial and subject, for each sample in the pre-stimulus interval, thus resulting in one SCP d.v. estimate every 100 ms. These are the SCP d.v.s that are plotted in Figure 2A and 2D.

Then, because our analysis goal was to correlate SCP d.v. with alpha/beta power estimates during the pre-stimulus interval, and because alpha/beta power had a lower temporal resolution, being estimated using 400-ms time windows, we computed the mean SCP d.v. for the four corresponding 400 ms time windows in the pre-stimulus period. This guaranteed we had the same temporal resolution for SCP d.v.s and alpha/beta power before conducting the correlation analysis (Fig 2C and 2F).

We have now added more details to our description of the SCP d.v. analysis on p.18. We also specify that an additional difference between the analysis of the two datasets is in the cross-validation approach, which involved 5-fold cross-validation for the low-level dataset, and leave-one-trial-out cross-validation for the high-level dataset. Although these choices should not make a practical difference in the results, we have chosen to keep them the same as prior studies to allows easier comparison between the current study and those prior findings. Finally, we want to emphasize that the aim of this study is not to formally compare the results between the low- and high-level datasets, but rather to conduct parallel analyses that ensure the reproducibility and generalizability of our main findings across visual stimuli and visual tasks.

The strategy depicted in Fig 1E is a schematic representation of the decoding approach for both the low-level and high-level datasets, which are identical except for relying on different classifiers. We have now clarified that the depiction is conceptual in the figure legend (rather than specific to the details of SVM or logistic regression).

Minor:
p.17 clustering analysis: time windows were treated independently so strictly a Bonferroni correction of 4 would need to be applied?

We thank the reviewer for this question. We chose not to conduct a Bonferroni (or another type of) multiple comparison correction across time windows because our goal with this analysis was to use a relatively liberal threshold for the inclusion of clusters. Indeed, our aim was not to ascertain that individual clusters in individual time windows showed a robust statistically significant effect, but rather, for any cluster with an effect for alpha or beta's influence on perception, to check the relation of this activity with SCP d.v. In this sense, we decided to rely on an inclusive strategy for identifying alpha/beta clusters, so that our analysis probing the relationship of pre-stimulus alpha/beta oscillation and SCP activity can be more comprehensive and thorough.

Please add to the citations:

On impact of LC activity on alpha rhythmic firing:
Dahl et al TICS (2022) Noradrenergic modulation of rhythmic neural activity shapes selective attention. DOI:https://doi.org/10.1016/j.tics.2021.10.009
On SCP influence on upcoming visual perception:
Glim et al Conscious Cogn (2020) The temporal evolution of pre-stimulus slow cortical potentials is associated with an upcoming stimulus' access to visual consciousness. DOI: 10.1016/j.concog.2020.102993

We thank the reviewer for these references, which we have now added to our manuscript, in the Discussion section.

Reviewer #2:
The authors investigate the extent to which prestimulus oscillatory and non-oscillatory activity (slow cortical potential, SCP) independently or jointly predict reports of visual perceptual awareness. The current project emphasizes the particular relation between aperiodic fluctuation and lower-frequency (alpha/beta-band) oscillatory power in the prestimulus signal. This question is rooted in a large body of work that has examined the relation between prestimulus oscillatory activity and subsequent perception, where the focus has typically been on either oscillatory or SCP activity in isolation (with little inquiry regarding how these dynamics intersect). The current project extends this investigation through examination of the association between the two measures (applied in two independent samples performing distinct visual tasks), as well as their relationship with pupil-linked arousal (as measured through pupil size). The association between aperiodic fluctuation and neural oscillation is likely of current interest to a broad cross-section of researchers in the field of cognitive neuroscience, and in principle, I have no objections to the question or main features of the analytic approach. I do have some concerns related to the implementation (highlighted below), but I believe these are answerable.

The following are separated into major/minor comments listed as far as possible in order of appearance in the manuscript. Those concerns that I think most essential to address are noted parenthetically with "In the reviewer's opinion, essential to address."

We thank the reviewer for the thoughtful and constructive comments.

Major concerns/questions:

Introduction:
Overall, the introduction is clear and well-written. However, there are a few areas that could benefit from more cautious writing. One general point is below, and a few specific notes are highlighted in the 'minor comments' section.

Introduction, Paragraph 2 and throughout the document:

The language choices of the authors regarding the relation between ongoing changes in EEG signal and perception are very causal. Although there certainly appear to be relations between oscillatory/low-frequency signal changes and perceptual variability, claims regarding degree of causality ('influences') are still active subjects of debate in the literature (as well as claims regarding which perceptual processes are impacted). The authors might consider adopting more cautious language surrounding the nature of the

relation between prestimulus activity and perception (perhaps a choice as simple as shifting from 'influence' to 'may influence').

We thank the reviewer for this suggestion; we have now gone through the text, and wherever possible, we have adjusted the language such that causal suggestions are minimized. We do so by saying that our results suggest a role of these neural activities in perception while being careful not to imply that our results can be taken as evidence of causal relationships. It is pretty self-evident that the present study did not employ causal manipulation. That said, the term "influence" is commonly used in studies investigating pre-stimulus neural activity's predictive power for behavior measured later in the trial (also see our reply to the reviewer's minor comments #1 below), and our choice of terminology is consistent with this long-standing literature.

Method & Results:
I apologize if my comments on method here seem lengthy. In part, this is due to the fact that the authors' main analytic goals depend on obtaining accurate representations of prestimulus SCP and oscillatory activity, so it seems important to consider any analytic choices/potential confounds that could impact measurement.

Eye movement/blink preprocessing:
The authors adopt the practice of removing eye movement-related artifacts with ICA, and rejecting trials in which blinks occurred during presentation. Both of these are standard and good-practice approaches to preprocessing in this context. However, I think the analyses here could be improved by including a more detailed assessment of blink-related activity.

I have concerns on two broad counts. First, residual blink artifacts in the MEG signal can remain after ICA and would likely be within the frequency range targeted by SCP analyses. For this reason, it would be useful to understand if & to what extent the presence of blinks in the pre-stimulus window impacts the size of the low-frequency effects (meaning, when trials with detectable blinks in the prestimulus analysis window are rejected, do differences related to subsequent behavioral response remain?).

Second, from both a cognitive and neural standpoint, there are ways in which blinks can impact subsequent awareness and neural activity beyond myogenic contamination of the MEG signal (see, for example, Kern et al., 2021 for an assessment of blink-related changes in intracranial occipital activity). It would therefore also be valuable to know whether frequency/timing of blinks in the prestimulus interval differs for 'seen' vs. 'unseen' targets (i.e. whether blink-related activity might reasonably explain either SCP or oscillatory effects, and possibly alter the apparent relation between them). This could be done for the second experiment most easily (given that eye-tracking data are not available for the first). This would also provide more context for the mediation results.

We thank the reviewer for pointing out these important points, which we address together.

The reviewer's questions concern the possibility of remaining blink artifacts in the MEG signal after ICA is conducted. A key feature of our pre-processing pipeline, which we acknowledge was not described clearly enough in our Methods section, is that after the ICA, we further clean the data for artifacts by removing any samples and trials in the MEG data that contained eyeblinks, based on the eye-tracking data. To do that, in the eye-tracking data, we identify blinks using our algorithm, in which we find time points where the diameter of the right eye pupil drops below 3.6 measurement units, and we define blink onset and

offset as 40 ms before and after crossing this threshold. Any timepoint in any trial in the MEG data that was affected by blinks was completely removed from further analysis. Given this approach, we are confident that our data do not contain any blink artifacts. We apologize that the relevant descriptions were not clear enough in our previous Methods section due to this step being already described in a previous paper using the same dataset (*7*). We have now clarified this processing pipeline in our Methods section on p. 16.

The frequency of blinks in the seen and unseen trials is shown below (calculated for the 2-sec pre-stimulus window). We note that although there is a difference in the frequency of blinks between the two conditions, the frequency of blinks overall is extremely low, with subjects having on average 0.027 blinks per trial in the Seen condition, and 0.01 blinks per trial in the Unseen condition. That is, on average, there is one blink in ~37 seen trials and ~100 unseen trials. Notwithstanding these differences, since we removed all trials with blinks from all further analyses, we are confident that any such differences between conditions would not affect our findings.

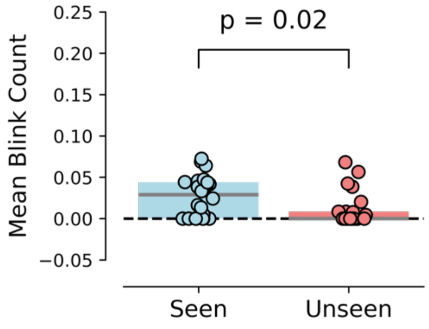

Wavelet decomposition:

The justification for choosing a wavelet approach to signal decomposition here is a bit unclear. Given that wavelets necessarily involve a degree of temporal smearing (which the authors do make an effort to avoid), an alternate approach would be to compute the Fourier transform over each prestimulus window. This would avoid issues of temporal smearing, and also avoid the issue that the shape of the power spectrum is not the same when averaging over wavelet-transformed output vs. taking the simple FFT of data in the same window—which can impact the results of the 1/f decomposition. It seems preferable here on all counts (avoiding temporal smearing, desiring to perform a 1/f decomposition of the data) to adopt an FFT-based approach. If the authors are concerned about the sampling resolution of the first dataset, zero-padding to the same resolution as the second dataset would allow approximate comparison of frequency windows between datasets.

For several reasons, we chose to use the wavelet decomposition method over FFT. First, wavelet decomposition is a standard approach used in multiple previous publications to obtain the power spectrum for the pre-stimulus period (*8-13*). This method has the advantage of having a higher temporal resolution and a lower frequency resolution for higher frequencies and, vice versa, a lower temporal resolution and a higher frequency resolution for lower frequencies (*14*). Thus, wavelet decomposition is adapted to the physiological reality of neurobiological signals, as signals with a higher frequency tend to change faster over time and, conversely, signals with a lower frequency change slowly over time. In addition, a fixed frequency interval (e.g., 5 Hz difference) makes a much larger physiological difference at lower frequencies (e.g., 3 vs. 8 Hz activity) than at higher frequencies (e.g., 65 vs. 70 Hz activity).

Second, as the reviewer pointed out, because we were aware of the potential caveat of temporal smearing, we ran a simulation to verify which time windows would be unaffected by any temporal smearing from the post-stimulus period. We then took the conservative approach by restricting our analyses to -1.7 to -0.1 s of the pre-stimulus interval, entirely avoiding the potential problematic time window (See Fig. S1). This approach is similar to other previous studies (*10, 15*).

Third, given that we were interested in obtaining several power spectra across the pre-stimulus interval, using 400-ms time windows, the minimum frequency resolution for FFT would be 1/0.4 s = 2.5 Hz due to the uncertainty principle. With the wavelet approach, we could obtain a much higher frequency resolution (0.8 Hz), resulting in more estimates within the alpha range and a lower minimum frequency. We think that the higher frequency resolution makes the wavelet approach more desirable than FFT in our case.

We have now further detailed our entire pre-processing and analytical pipeline in a new Supplementary Note.

Decomposition using Fooof & selection of trials (p.17; In the reviewer's opinion, essential to address): Theories regarding prestimulus activity fluctuations and the degree to which they do/do not predict subsequent reports of visual awareness acknowledge that oscillatory power can shift dramatically from trial to trial. These shifts are part of what is hypothesized to lead to changes in subsequent awareness. Accordingly, it doesn't seem justified to exclude a trial/timepoint simply because Fooof does not discover a peak for the spectrum in that window (this also seems problematic from a statistical perspective when trying to understand the relations between signals). Rather, if the authors are inclined to use 1/f decomposition, it seems like a safer approach would be to obtain more stable estimates of center frequency and bandwidth by deriving them from the overall signal for each electrode/subject/task. Then, apply these to the single-trial, 1/f-corrected spectra, ultimately obtaining even numbers of measurements for beta and alpha power for each timepoint/trial (i.e., avoiding variation in the number of trials/electrodes contributing to alpha-band, beta-band and SCP measurement).

Below, we explain our rationale for the decomposition method and selection of trials. Our goal was both to obtain oscillatory estimates for as many trials as possible, and to only take estimates for oscillatory peaks when we had some confidence that a peak actually occurred. When we first ran our analysis with the default fooof settings, we noticed that the number of trials in which fooof did not identify an oscillatory peak was not insignificant. Indeed, previous work has shown that oscillations come and go (*16, 17*), and hence do not necessarily occur in every trial. For instance, alpha power is known to have large inter-trial variability, especially in tasks that involve visual attention such as this one (*18*) or those with a high cognitive load (*19*). Similarly, converging recent research showed that beta oscillations are transient signals that occur in 'bursts' (*20*) and hence cannot be expected to be present in every trial. Since we cannot assume that alpha or beta oscillations occur on each trial from a principled perspective, adopting the method suggested by the reviewer would erroneously attribute an oscillation to every trial even when no oscillation occurs on a particular trial. In addition, our approach allows the center frequency of alpha or beta oscillation to vary across trials, which is consistent with a large body of prior work showing that center frequencies of brain oscillations vary over time within an individual [e.g., (*21-23*)].

On a more technical point, we note that our input power spectra for fooof were obtained from single trial epochs and were hence relatively noisy. Thus, if we assumed that there was an alpha or beta peak in every trial, the fooof algorithm would take for peaks what was actually noise. Therefore, we chose a middle

ground between being too conservative (sticking to fooof's default settings of 2 SD for peak identification, which is appropriate for very robust, averaged power spectra) and being too liberal (assuming there was a peak in every trial, and running the risk of identifying a noise variation as an oscillation). To this end, we changed the peak detection parameter in fooof. The default parameter is to mark as a peak any fluctuation that is 2 standard deviations above the mean of the entire power spectrum. We changed this threshold to 1 standard deviation and obtained alpha peaks in 88 % of trials and beta peaks in 79.4 % of trials.

In the process of deciding on this analysis pipeline, we also performed a control analysis, which was omitted from our original manuscript. In this analysis, we compared all trials with an alpha peak to all trials without an alpha peak, in terms of behavioral measures, beta power, and SCP d.v. We also compared all trials with a beta peak to all trials without a beta peak in terms of behavioral measures, alpha power, and SCP d.v. For both sets of comparisons, we found no significant differences in behavior or neural measures. In the figure below, we present one representative cluster for both alpha and beta in the low- and high-level dataset; for all sensors in the cluster, we identified trials with and without an oscillatory peak, and compute the average detection rate, SCP d.v., and oscillatory power. The two groups of trials were compared using a Wilcoxon signed rank test. These results show that the selection of trials with a peak are representative of the entire sample in terms of the behavioral and neural metrics.

We have now added to the manuscript percentage of trials considered to contain an oscillation and clarified that these trials were found to be representative of the entire sample in this control analysis (p. 17).

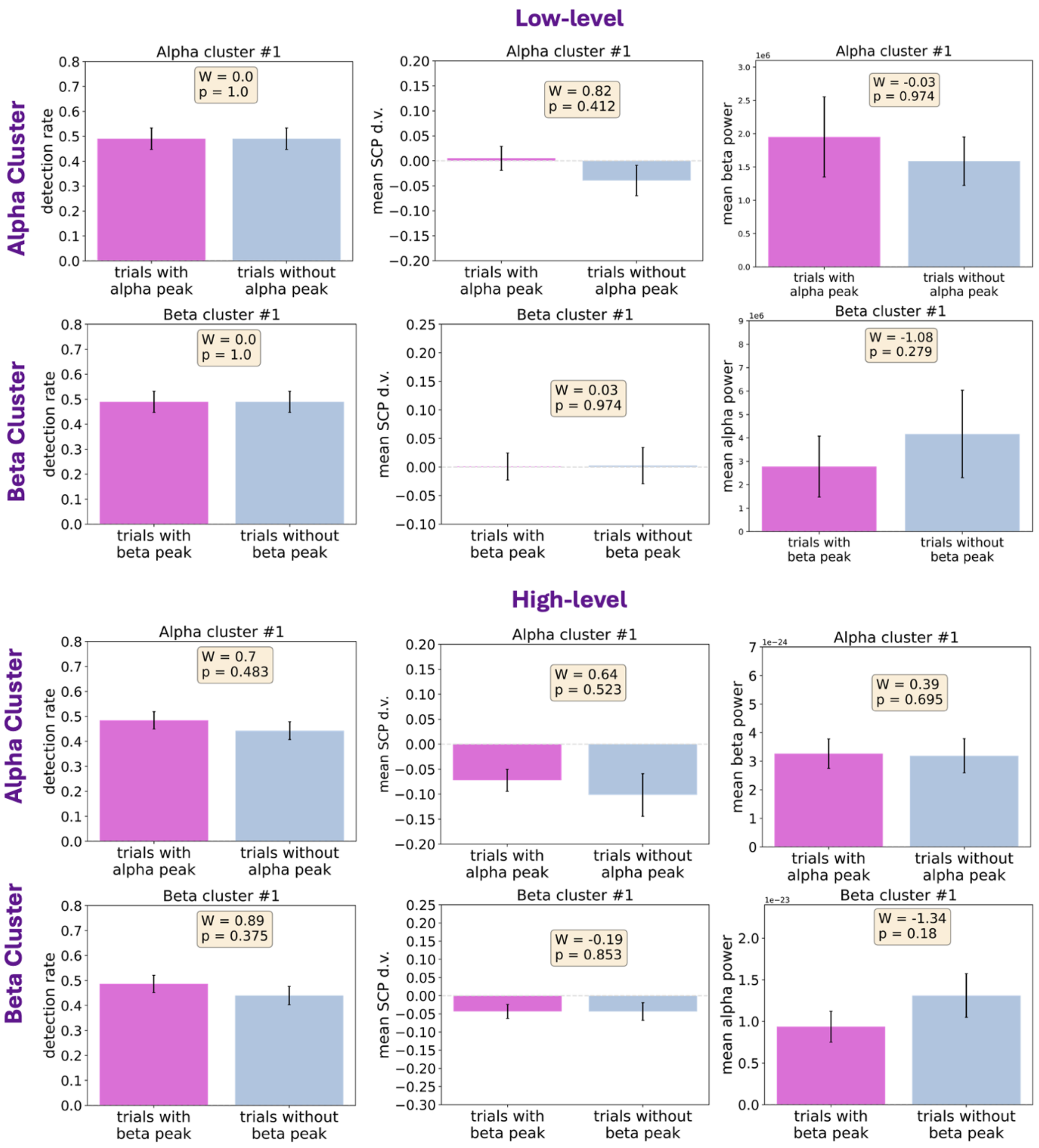

Relatedly, would the authors consider including a breakdown of how many trials remain for analysis for each subject/condition after all exclusions are performed? In addition, if the authors are disinclined to follow the suggestion above, it would be helpful to include information regarding the lack of inclusion of non-oscillatory trials in the main text of the document, since this is important to evaluation of the main analyses.

We obtained alpha peaks in 88% of trials and beta peaks in 79.4% of trials. We have now added this information to p. 17 of the manuscript.

Correction for 1/f activity in log vs. linear space (In the reviewer's opinion, essential to address): It is not entirely clear from the method section whether the authors perform 1/f correction in log space (the Fooof default) or linear space (after back-transforming aperiodic estimates into linear space). Each of these approaches implies different assumptions regarding how the sources of oscillatory and aperiodic data are combined at the scalp level, and if these assumptions are violated, the relation between aperiodic and oscillatory activity can be warped as a result. Since the primary aim of this paper is to relate changes in a low-frequency aperiodic pattern to changes in oscillatory power, it seems critically important to consider and explicitly justify these assumptions. To elaborate (perhaps too much):

Most individuals attempting separation of broadband and oscillatory activity assume, implicitly or explicitly, that the generative process is additive - that is, that broadband and oscillatory time series are additive at the level of the scalp (this is consistent with available evidence; e.g. Gyurkovics et al., 2021, among others). Parsing a power spectrum into oscillatory and non-oscillatory components then relies on the linearity property of the fourier transform, i.e. the spectrum of the sum of two signals is equal to the sum of their respective spectra (spectrum(oscillation + broadband) = spectrum(oscillation) + spectrum(broadband)). This implies that one can simply subtract the broadband spectrum from the aggregate spectrum to obtain a reasonably accurate impression of the spectrum of an oscillation.

However, many approaches to spectral separation (including Fooof) encourage separation of spectra in log or semi-log space, adopting a subtractive approach to decomposition. The logic is often presented as some form of: log(oscillation_spectrum + broadband_spectrum) - log(fitted_broadband_spectrum) = log(oscillation). But the additivity of spectra is not preserved over log transformation (i.e., log(a+b) != log(a) + log(b), and correspondingly, log(a+b) - log(b) != log(a)). If the generative process actually is additive, obtaining an accurate understanding of 'oscillation_spectrum' and 'broadband_spectrum' cannot be achieved by applying a subtractive correction in log space. Rather, subtraction in log-space is only justified if there is reason to believe that the generative process is multiplicative (i.e. log(oscillation*broadband) - log(broadband) = log(oscillation)).

This is not just a theoretical issue. In our own data, we've seen adjustment in log-space result in dramatically different oscillatory effects (& relations between oscillatory power estimates and aperiodic fluctuation) than adjustment in linear space. We suspect these issues have contributed to a number of reports currently in the literature.

Here, it appears that the authors have (understandably, given the literature) adopted a log-space subtraction approach. If the authors believe multiplicative generation is the source of their spectra, this should be explicitly stated & justified. Otherwise, the correction approach should be modified to correct in linear space.

We thank the reviewer for raising this important point. We agree on the importance of this issue, and we explain our analysis choices and their rationale below.

We have now added a Supplementary Note, in which we explain the entire analysis pipeline in detail, with plots illustrating every step. Specifically, we input power spectra into fooof, which we obtain from a wavelet algorithm and are in linear space. Fooof then takes the linear power spectrum, converts it to log

space, and computes a broadband fit. It then subtracts the broadband fit, which is in log space, from the original, log-space power spectrum, to obtain a flattened spectrum. We note here that subtraction in log space is used by the algorithm, hence assuming an underlying multiplicative relationship between the aperiodic and the oscillatory spectra as pointed out by the reviewer.

On this flattened spectrum, it computes an oscillatory fit by fitting a series of Gaussians. The fooof algorithm then outputs the following: a log-space broadband fit, a log-space full power spectrum fit (which includes both oscillations and broadband fit), and a list of oscillatory peaks, including the power (in log space), central frequency and bandwidth. Importantly, we have verified that the Gaussian fits carried out by fooof does a good job of identifying and estimating the peaks in the power spectrum, despite the initial subtraction carried out in the log space. And also critically, we do not use fooof's estimates of the oscillatory peak power. Instead, we adopted a different approach that assumes a linear summation of oscillatory power and 1/f power, as detailed below.

Our goal was to have power estimates of the oscillatory peaks that took into account both the height of the oscillation above the aperiodic fit, as well as the bandwidth of the oscillation. To do that, we computed the area under the curve (AUC) of each oscillatory peak. We first converted both the aperiodic spectrum and the full power spectrum, which are output by fooof, into linear space. We then use the oscillatory peak frequency and the oscillatory bandwidth output by fooof (which are both in Hz) and used these to compute the AUC. Specifically, we took the sum of linear power from one bandwidth below the oscillation's center frequency, to one bandwidth above the center frequency, for both the full model fit (aperiodic and oscillatory) and for the aperiodic fit. We note that we used a range of two bandwidths in order to capture the entire oscillatory peak, given that the bandwidth output by *fooof* is the full-width-at-half-maximum, and thus captures only the 'top half' of the peak. We thus obtained the AUC for the broadband fit and the AUC for the full model fit within either the alpha or the beta band. To get the power estimate for either oscillation, we then perform a subtraction of the full model fit AUC minus the broadband fit AUC, both of which are in linear space. This step yielded our oscillatory power estimates, which we used as input for our clustering analysis, and for all further analyses.

In this way, the subtraction we used to obtain oscillatory estimates occurred in linear space, and our analysis does assume a linear summation between oscillatory power and 1/f power in the full power spectrum. The fooof procedure was mainly adopted to perform the Gaussian fit to the oscillatory peaks, which, as we mentioned earlier, appears to do a decent job. Indeed, we settled on this procedure in our original analysis as we believe that the linear summation assumption makes more theoretical sense (*24*).

We have explained the entire procedure in detail in a newly added Supplementary Note, and we would like to thank the reviewer once again for raising this important issue, which prompted us to clarify our analysis procedures.

Merging 'real'/'catch' trials (Results, p4):
The choice to merge both real/catch trials (and both intact/scrambled image trials) is a bit unusual. It seems like it would be more appropriate to separately examine target-present trials (first dataset) and intact images (second dataset). In both cases, the choice to merge trials from both conditions means that what is actually measured is not really whether prestimulus power predicts perceptual experience, but rather the likelihood of making a 'seen' response regardless of stimulus. In the case of the first dataset, there are so few catch trials that the decision to include them or not may not ultimately impact the conclusions drawn here. However, in the second case, 60 scrambled 'catch' trials is not an insignificant

number. Given that there is evidence that pre-stimulus activity (particularly in the alpha range) may impact some aspects of decision-making but not others (e.g. Iemi et al., 2017; Samaha et al., 2020) it seems very inadvisable to lump 'hits' and 'false alarms' into the same data pool without interrogation. If the only justification for pooling this data is 'statistical power', then there should be some demonstration that the pattern of results does not qualitatively differ between intact/scrambled images.

We thank the reviewer for this point. We have explained the rationale for including both 'real' and 'catch'/'scrambled' trials in our response to Reviewer 1, point 1, in detail, which we would like to refer the reviewer to. We have now conducted an additional control analysis, in which we show that the decision axis for decoding recognized and unrecognized trials in the high-level dataset based on all trials (i.e., including both real and scrambled) is very similar to the decision axis for decoding recognized and unrecognized trials in only real trials. In addition, our earlier analysis using the high-level data set had shown that the decoder trained to predict "recognized" vs. "unrecognized" responses could cross-generalize between real and scrambled trials (Fig. 2A in Ref (*3*)). Therefore, we are confident that the inclusion of the scrambled trials (which make up 16.7 % of trials in the high-level dataset, and only 5% of trials in the low-level dataset) does not significantly change our results.

In addition, the claim "As such, we focus on whether different pre-stimulus neural activities have shared influences on perceptual outcome, instead of dissociating pre-stimulus activity's influence on sensitivity and criterion related to conscious perception as previous studies have done." However, the choice to pool hits/false alarms into a single category essentially means that the authors are examining effects likely related to criterion.

We agree with the reviewer's assessment that we are likely examining effects related to the criterion. This interpretation is consistent with the earlier work that the current study builds on, showing that both pre-stimulus SCP and alpha power correlate with the criterion of conscious perception (*1, 3*). The crucial difference, here, is that to examine the criterion directly, we would need to pool trials together, but the focus of our study is on variability across individual trials, which is not conducive for SDT analyses. Nonetheless, we explicitly mention that our results correspond to the subjective experience of detection (low-level dataset) or recognition (high-level dataset), regardless of whether a stimulus is present or not, and hence the effect is akin to a criterion effect.

SCP vs. oscillatory analyses:
For analyses of SCP and pre-stimulus power, I am not sure I follow why a classification approach over all electrodes is adopted for the SCP data, and a simple power comparison is conducted for alpha and beta-power analyses over a smaller range of timepoints. To maximize capacity to relate SCP dynamics to higher-frequency oscillatory power dynamics, I would think it preferable to maximize similarity of analytic approaches here. Perhaps I'm missing some feature of the analysis that necessitates this approach. Could the authors clarify why they have adopted two distinct approaches here?

We thank the reviewer for this question. Our analysis choices for extracting perceptually relevant pre-stimulus SCP activity and oscillatory power were motivated by both prior literature and the nature of these signals. For brain oscillations, increased power has an unambiguous meaning—namely, that the oscillations are stronger and the peak-to-peak amplitude is larger. Previous studies have shown that increased or decreased alpha or beta power over spatially localized brain regions carries functional significance as it indicates the wax-and-wane of the underlying oscillation's strength (*1, 25, 26*). This is

why our approach of cluster-based correction in the sensor space, which focuses on spatially contiguous groups of sensors with a similar direction of power change in relation to perceptual outcome, makes sense for oscillatory power. In addition, to achieve a stable estimate of power, we used 400-ms time windows.

By contrast, SCP's functional significance manifests not via its power, but via its raw amplitude fluctuations in the filtered, non-rectified signal (*27-29*). This is akin to phase information, although phase is classically defined for an oscillatory signal (*30*). For EEG recordings, the negative-going phase of surface-related potentials tends to indicate a more excited cortical state (*27-29, 31, 32*). But for MEG recordings, the sign of the phase becomes arbitrary because it depends on where the sensor is situated spatially in relation to the underlying source. Therefore, for MEG studies such as ours, it is the large-scale spatial *pattern* of the SCP signals that are predictive of perception, as revealed by our earlier studies (*2, 3, 6*). Accordingly, we use a classifier over the whole-brain MEG signals to extract perceptually relevant pre-stimulus SCP activity; and because this signal has already been filtered in the frequency domain, an arbitrary time resolution can be used for this analysis.

Correlation analyses:
This is optional, but to better capture the hierarchical structure of the data (association between trial-wise alpha power and SCP, for example, nested within subject), it might be useful here to generate mixed-effects models with subject as a random effect (instead of correlating within subject and then performing a group-wise analysis over the correlation coefficients).

We agree that running a mixed-effects model with subject as a random effect would be an informative analysis. However, we believe that carrying out such an analysis would be beyond the scope of this paper.

Interpretation of Correlations & Bayesian tests (p6; In the reviewer's opinion, essential to address):
The interpretation of Bayes Factors here is a bit unconventional (although this seems to be an issue associated with the correlation analyses and not the rest of the analyses in the document). A Bayes Factor of 1 corresponds to ambiguous evidence for/against either of two hypotheses. Although criteria for evaluating magnitude of Bayes Factors are somewhat arbitrary, the convention in much of the literature is to treat Bayes Factors of between ⅓ and 3 as providing weak/ambiguous evidence. So, a Bayes Factor of .828 (very nearly 1) corresponds to at best ambiguous evidence very weakly favoring the null hypothesis of no relation. It should not be considered moderate evidence, and certainly doesn't suggest a lack of correlation. Similarly, a Bayes Factor of .331 would most often be considered weak evidence in favor of the null hypothesis, and a BF of 1.24 would probably best be considered essentially ambiguous.

We apologize for the unconventional interpretation of the Bayes Factors. We have now updated the text in the correlation analysis results section to follow the convention suggested by the reviewer.

Alpha power & SCP, paragraph 4 - "Together these results suggest" - The authors might want to revisit the conclusions in this paragraph, which do not reflect the evidence just provided. Phrases like "did not co-vary" and "must be independent" are too strong. A non-significant p-value does not imply a total lack of shared variance, and the Bayes Factors provide at best weak/ambiguous evidence in either direction. It would be more accurate to say something like "although each spontaneous signal was individually linked to subsequent behavior, there was not evidence of a strong association between the signals." `

We have now updated the conclusions for this section on p.6 in line with the reviewer’s suggestion.

Beta power and SCP, paragraph 2 - "This result suggests" - As above, it is incorrect to claim that a lack of significant correlation implies independence, or that these results imply that these two signals "do not have any covariation."

We have now updated the text in line with this suggestion.

Beta power and SCP, paragraph 3: The authors are correct that Bayes Factors of 1.835 and 2.777 provide at best weak evidence in favor of the alternative hypothesis (for some additional elaboration on Bayes Factor interpretation, see Kass & Raftery, 1995). As such, I don't think it's entirely accurate to use these Bayes Factors to support the statement that "the correlations between beta power and SCP d.v. were different from 0" (this statement is maybe better placed in reference to the initial p-values).

We have now removed this sentence.

Discussion:
As with the introduction, the discussion is generally clear and well-written. I have just one comment on language use that I think could improve the document. In paragraph 1 (and throughout), the authors repeatedly make claims about independence that seem too strong/incautious relative to the data presented. The data might support claims about possible or partial independence of mechanism, but to imply that oscillatory and aperiodic activity patterns reflect totally independent mechanisms linked to perception is a bit beyond what the data support. The authors might consider re-evaluating the discussion section with an eye toward more careful language.

Thank you for this comment. We have now updated the language in the Discussion in line with the reviewer’s suggestion.

Minor comments:

Abstract: This is relatively minor, but the statement 'We found that oscillatory power and large-scale SCP activity influence conscious perception through independent mechanisms that do not have shared variance' could be written more cautiously given the design. Use of 'influences' implies a known causal brain-behavior relation that is not clearly warranted given the literature and the correlational structure of the data. Second, and perhaps more importantly, 'do not have shared variance' is probably too strong given the results, which don't imply a totally orthogonal relationship between the two signal features.

We agree with the reviewer that our study did not employ causal manipulation, but the word “influence” has been used in many prior studies on pre-stimulus effects that predict behavior in a statistical sense [e.g., (*1, 33, 34*)]. We believe that our choice of terminology is in keeping with this tradition, and the fact that the present study is a correlational study is pretty self-evident. In terms of shared variance, we believe that our findings indeed suggest that there is negligible shared variance between SCP and oscillation power in their prediction of perceptual outcome (see our reply to the reviewer’s point “beta power and SCP, final paragraph” below).

Introduction, paragraph1, sentence 3: The authors might consider revisiting the two examples provided, as they are not clear examples of the argument 'This variability shows that conscious perception is not determined solely by the incoming sensory information.' Variability in perception is probably better highlighted as variability in experience of the same physical stimulus under different circumstances. The current examples are of two very different stimuli, and it could easily be argued that the stop sign is more salient to a driver than an existing crack in the windshield (which might not be salient at all to a driver used to its presence). This is not a strictly necessary change, but if the language distracted this reviewer, it seems likely to distract other readers too.

We thank the reviewer for this thoughtful comment; we agree that the examples used might not have been the best because they focused on different types of sensory information that could explain the different percepts we discussed. We have updated the example provided, which does a better job of highlighting the perceptual variability we are interested in.

Introduction, paragraph 3: 'perceived or missed' - the authors might consider revisiting the choice of 'perceived' here, since although alpha power dynamics seem to predict subjective reports of awareness, the evidence that they actually predict the subsequent percept is at best debatable (e.g. Iemi et al., 2017).

We respectfully disagree with the reviewer's interpretation of the Iemi 2017 study (we assume that this is the J Neurosci paper). This study showed that pre-stimulus alpha power influences criterion but not sensitivity of conscious detection. As the author explained in detail in a follow-up review (Samaha, Iemi et al TiCS 2020), this criterion effect most likely relates to subjects' conscious perception and not just a report mechanism, which is consistent with occipital alpha power being associated with sensory cortical excitability (instead of downstream reporting mechanisms). More broadly, changes in conscious perception can result from a change in sensitivity or a change in criterion. This interpretation by Samaha, Iemi et al is consistent with our own interpretation of SCP's influence on the criterion component of conscious recognition in the Podvalny et al. 2019 study.

Method, EEG preprocessing (both datasets): This is optional, but it would be helpful for the reader if the data preprocessing (or results section in the main document) included a statement regarding the extent to which preprocessing/analysis steps were identical/differed between these analyses and prior published studies with these datasets.

We thank the reviewer for this comment. We have now added a paragraph on p. 18 that details the similarity and differences between the analysis pipelines applied to both datasets.

Classification analyses: Could the authors clarify the rationale for applying two distinct approaches to classification for the low- and high-level datasets? It seems preferable when looking across two datasets in the same paper to apply the same approach in both cases as far as possible, although admittedly not necessary since datasets are treated independently throughout.

Our decision to use different classifiers for the two datasets was based on the motivation to extend the results of the previous studies that had been published with these data. The Podvalny et al. 2019 study

(*3*) used logistic regression classifiers because they yield a probability-based interpretation that can directly be compared with our hypotheses in that study (see Methods therein). Therefore, we opted to use the same classifiers to build on previous findings here. We have detailed this methodological consideration in the decoding section of the Methods section. (A more detailed discussion of this point is also provided in response to Reviewer #1's major point 2.)

1/f decomposition: The figure illustrating aperiodic decomposition appears remarkably similar to one used by the authors of Fooof on their website (https://fooof-tools.github.io/fooof/auto_examples/plots/plot_fooof_models.html#sphx-glr-auto-examples-plots-plot-fooof-models-py). If this figure was inspired by or adapted from their work, it seems warranted to credit them in the caption.

Thank you for pointing out this missing reference. The figure is indeed adapted from the *fooof* toolbox. We have updated the Figure legend for Figure 1 to credit the toolbox and its authors.

Results, Paragraph 4 of 'Pre-stimulus alpha power predicts perceptual outcome': The authors might consider discussing how these results (in the higher level dataset) could relate to prior observations in the literature of differing pre-stimulus dynamics related to responses in detection vs. discrimination contexts (e.g. Iemi et al., 2017).

We understand the reviewer's point, but we believe that the object recognition probed by our high-level task is quite different from the discrimination task in Iemi et al. 2017. Specifically, our object recognition task is a detection task, but at a higher level (detecting a meaningful content). The categorization question in the high-level dataset or the orientation discrimination question in the low-level dataset (not analyzed here, but reported in the original papers (*3, 6*)) would be more similar to the discrimination response probed in Iemi et al. Therefore, we believe that adding the point the reviewer suggested might cause more confusion.

Figure 2: If possible, it would be helpful to have some sense of the topographic distribution of prestimulus SCP activity in both tasks, so as to better relate this to the observed clusters of alpha/beta power.

Below, we plot the activation patterns for pre-stimulus SCP activity in both the low-level and the high-level datasets. Because these findings have been reported in prior publications (*2, 3*), we do not reproduce them in the main figures of the present paper.

Low-level dataset activation pattern:

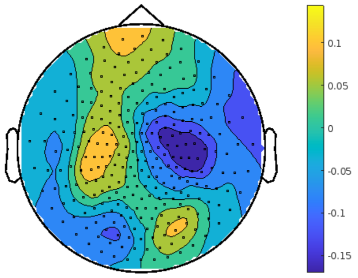

High-level dataset activation pattern:

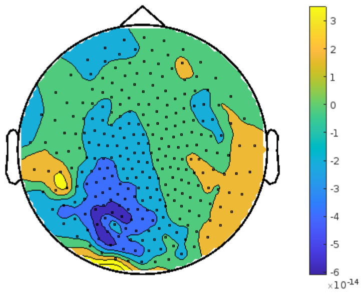

Beta power and SCP, final paragraph: If I understand the AUROC analyses correctly, the statement "This demonstrates that pre-stimulus beta power and SCP d.v. explain non-overlapping variance in the perceptual outcome" may not be strictly correct. These analyses demonstrate that non-shared components of variation in beta power and SCP can each predict subsequent perceptual outcome, but I don't think this necessarily implies that the variance in perceptual outcome that they both predict is itself non-overlapping.

We disagree with the reviewer’s interpretation here. Because SCP’s predictive power for perceptual outcome after regressing out the component of SCP that correlates with beta power is unchanged, and, vice versa, this result does suggest that the two pre-stimulus signals explain non-overlapping components of the variance in perceptual outcome. This is because otherwise, regressing one signal from the other should produce an appreciable decrease in its predictive power for perceptual outcome, but our results do not even show a trend for this effect (Fig. 4B-C).

Discussion, general: The authors might consider discussion of how their results relate to the observations of Gyurkovics et al. 2021 (The impact of 1/f activity and baseline correction on the results and interpretation of time-frequency analyses of EEG/MEG data: A cautionary tale) that alpha-band power is not positively correlated with 1/f 'background' (See also single-trial correlational results reported by Cunningham et al., 2023). 1/f slope and offset in the EEG can be influenced by amplitude of low-frequency activity, so it seems worth acknowledging or considering how these existing results might or might not reflect similar observations to those described here.

We thank the reviewer for this important reference. We agree that this paper is highly relevant to our results. It highlights the issue of assuming a multiplicative relationship between oscillatory and broadband power, which often arises from decibel conversions. Their results are indicative that, in fact, the relationship between broadband and oscillatory activity is likely to be additive and hence operate independently. This aligns with our current analysis approach (Supplementary Note) and results, which show no evidence that oscillatory and broadband activities are correlated in predicting an upcoming perceptual outcome, consistent with an additive relationship. We now refer to this paper in our Discussion section, on p. 11.

Discussion P1: 'Importantly, our main findings were reproduced in two separate datasets involving different visual stimuli, showing the reproducibility and generalizability of the present findings.' I think this statement might be more misleading than the authors intend, since it seems to refer to their prior statement in this paragraph regarding pupil-linked arousal (the statement seems to suggest both pupil-linked and 'independence of signal' findings were fully generalized across two datasets). The authors might consider tweaking the wording to emphasize that 'main findings' refers to the relation between oscillatory power and SCP.

We thank the reviewer for pointing out this potential confusion. We have now updated the relevant sentence in the Discussion to emphasize which main finding we were referring to.

Reviewer #3:
Thank you for inviting me to review this manuscript by Koenig and He, in which the authors interrogate the relationship between low and high frequency MEG signals and two distinct threshold detection paradigms. Using a decoding-based approach, the authors demonstrate independent evidence for low and high frequency signals prior to stimulus presentation, which they then relate to the ascending arousal system through a statistical relationship with pupil diameter.

This study was well-motivated, clearly-presented and discussed within the context of a broad literature. I have only minor comments, which I hope are helpful.

* P4 - I'm not familiar with the term "temporal spearing". Please adjust or define.

The term “temporal smearing” refers to the spreading of information from one time point to adjacent time points in the wavelet-transformed signal. This smearing can cause signal from the post-stimulus period to leak into the pre-stimulus period due to the way wavelet analysis fits the data with wavelet functions across time. We have now defined this term in more detail on p. 16.

* Methods - as written, I worry that the reader may get the impression that statistical decodability is synonymous with conscious perception, whereas it's possible that signals were present in the pre-stimulus window that were related to the primary processing, but not the conscious perception of the stimuli. In my opinion, the manuscript would benefit from this point being made clearly for the reader.

We have now added a sentence on p. 19 to specifically preempt this potential misunderstanding.

* P10 - is it possible to create a model that combines alpha, beta and SCP with pupil size to determine their unique/combined perceptual influences?

We thank the reviewer for this suggestion. As it stands, we already include 3 variables at a time in our mediation models, e.g., SCP, pupil size, and perceptual outcome or alpha power, pupil size and perceptual outcome. Including all of the variables in one overarching model, with alpha power, beta power, SCP, pupil size and perceptual outcome, would result in a 5-variable model, and this would make the hypothesis space very large, with the number of permutations between variables very high. We therefore think that this model would be overly complex and therefore not very informative.

We would like to thank all three reviewers again for the thoughtful feedback, which has allowed us to significantly strengthen our manuscript.

References

1. J. Samaha, L. Iemi, S. Haegens, N. A. Busch, Spontaneous Brain Oscillations and Perceptual Decision-Making. *Trends Cogn Sci* **24**, 639-653 (2020).
2. A. T. Baria, B. Maniscalco, B. J. He, Initial-state-dependent, robust, transient neural dynamics encode conscious visual perception. *PLoS Comput Biol* **13**, e1005806 (2017).
3. E. Podvalny, M. W. Flounders, L. E. King, T. Holroyd, B. J. He, A dual role of prestimulus spontaneous neural activity in visual object recognition. *Nature communications* **10**, 3910 (2019).
4. S. Dehaene, H. Lau, S. Kouider, What is consciousness, and could machines have it? *Science* **358**, 486-492 (2017).
5. D. Rosenthal, Consciousness and confidence. *Neuropsychologia* **128**, 255-265 (2019).
6. Q. Li, Z. Hill, B. J. He, Spatiotemporal dissociation of brain activity underlying subjective awareness, objective performance and confidence. *J Neurosci* **34**, 4382-4395 (2014).
7. E. Podvalny, L. E. King, B. J. He, Spectral signature and behavioral consequence of spontaneous shifts of pupil-linked arousal in human. *eLife* **10**, (2021).
8. V. Wyart, C. Tallon-Baudry, How ongoing fluctuations in human visual cortex predict perceptual awareness: baseline shift versus decision bias. *J Neurosci* **29**, 8715-8725 (2009).
9. G. Buzsaki, *Rhythms of the Brain*. (Oxford University Press, New York, 2006).
10. L. Iemi, M. Chaumon, S. M. Crouzet, N. A. Busch, Spontaneous Neural Oscillations Bias Perception by Modulating Baseline Excitability. *J Neurosci* **37**, 807-819 (2017).
11. L. Iemi *et al.*, Ongoing neural oscillations influence behavior and sensory representations by suppressing neuronal excitability. *NeuroImage* **247**, 118746 (2022).
12. L. H. Arnal, K. B. Doelling, D. Poeppel, Delta–beta coupled oscillations underlie temporal prediction accuracy. *Cerebral Cortex* **25**, 3077-3085 (2015).
13. B. Han, R. VanRullen, The rhythms of predictive coding? Pre-stimulus phase modulates the influence of shape perception on luminance judgments. *Scientific reports* **7**, 43573 (2017).
14. J. Sinkkonen, H. Tiitinen, R. Näätänen, Gabor filters: an informative way for analysing event-related brain activity. *Journal of neuroscience methods* **56**, 99-104 (1995).
15. L. Iemi *et al.*, Multiple mechanisms link prestimulus neural oscillations to sensory responses. *eLife* **8**, (2019).
16. G. Buzsaki. (Oxford University Press, New York, NY, 2006), pp. 111-135.
17. G. Buzsaki, A. Draguhn, Neuronal oscillations in cortical networks. *Science* **304**, 1926-1929 (2004).
18. H. van Dijk, J. M. Schoffelen, R. Oostenveld, O. Jensen, Prestimulus oscillatory activity in the alpha band predicts visual discrimination ability. *J Neurosci* **28**, 1816-1823 (2008).
19. S. Palva, J. M. Palva, New vistas for alpha-frequency band oscillations. *Trends Neurosci* **30**, 150-158 (2007).
20. M. Lundqvist, E. K. Miller, J. Nordmark, J. Liljefors, P. Herman, Beta: bursts of cognition. *Trends in Cognitive Sciences*, (2024).
21. T. Ro, Alpha Oscillations and Feedback Processing in Visual Cortex for Conscious Perception. *J Cogn Neurosci* **31**, 948-960 (2019).
22. S. Katyal, S. He, B. He, S. A. Engel, Frequency of alpha oscillation predicts individual differences in perceptual stability during binocular rivalry. *Hum Brain Mapp* **40**, 2422-2433 (2019).
23. A. Wutz, D. Melcher, J. Samaha, Frequency modulation of neural oscillations according to visual task demands. *Proc Natl Acad Sci U S A* **115**, 1346-1351 (2018).
24. B. J. He, Scale-free brain activity: past, present, and future. *Trends Cogn Sci* **18**, 480-487 (2014).

25. O. Jensen, M. Bonnefond, R. VanRullen, An oscillatory mechanism for prioritizing salient unattended stimuli. *Trends Cogn Sci* **16**, 200-206 (2012).
26. B. Spitzer, S. Haegens, Beyond the Status Quo: A Role for Beta Oscillations in Endogenous Content (Re)Activation. *eNeuro* **4**, (2017).
27. N. Birbaumer, T. Elbert, A. G. Canavan, B. Rockstroh, Slow potentials of the cerebral cortex and behavior. *Physiol Rev* **70**, 1-41 (1990).
28. B. J. He, M. E. Raichle, The fMRI signal, slow cortical potential and consciousness. *Trends Cogn Sci* **13**, 302-309 (2009).
29. B. Rockstroh, T. Elbert, A. Canavan, W. Lutzenberger, N. Birbaumer, *Slow Brain Potentials and Behaviour*. (Urban & Schwarzenberg, München, Germany, ed. 2, 1989).
30. B. J. He, J. M. Zempel, Average is optimal: an inverted-U relationship between trial-to-trial brain activity and behavioral performance. *PLoS Comput Biol* **9**, e1003348 (2013).
31. P. Khader, T. Schicke, B. Röder, F. Rösler, On the relationship between slow cortical potentials and BOLD signal changes in humans. *International Journal of Psychophysiology* **67**, 252-261 (2008).
32. M. Devrim, T. Demiralp, A. Kurt, I. Yucesir, Slow cortical potential shifts modulate the sensory threshold in human visual system. *Neurosci Lett* **270**, 17-20 (1999).
33. S. Haegens, B. F. Handel, O. Jensen, Top-down controlled alpha band activity in somatosensory areas determines behavioral performance in a discrimination task. *J Neurosci* **31**, 5197-5204 (2011).
34. L. Iemi, N. A. Busch, Moment-to-Moment Fluctuations in Neuronal Excitability Bias Subjective Perception Rather than Strategic Decision-Making. *eNeuro* **5**, (2018).
